# Prevalence and characteristics of somatic symptom disorder in the elderly in a community-based population: a large-scale cross-sectional study in China

**DOI:** 10.1186/s12888-022-03907-1

**Published:** 2022-04-12

**Authors:** Yani Wu, Zhengyu Tao, Yongxia Qiao, Yezi Chai, Qiming Liu, Qifan Lu, Hongmei Zhou, Shiguang Li, Jialiang Mao, Meng Jiang, Jun Pu

**Affiliations:** 1 School of Public Health, School of Medicine, Shanghai Jiao Tong University, Shanghai, 200025 China; 2 Division of Cardiology, Key Laboratory of Coronary Heart Disease, School of Medicine, Shanghai Municipal Education Commission, Renji Hospital, Shanghai Jiao Tong University, Shanghai, 200127 China; 3 Division of Cardiology, Anhui No. 2 Provincial People’s Hospital, Anhui, 230011 China

**Keywords:** Somatic symptom disorder, Elderly, Epidemiology, Depressive disorder, Anxiety disorder

## Abstract

**Introduction and objectives:**

The aging population is expected to reach 2 billion by 2050, but the impact of somatic symptom disorder (SSD) on the elderly has been insufficiently addressed. We aimed to clarify the prevalence of SSD in China and to identify physical and psychological differences between the elderly and non-elderly.

**Methods:**

In this prospective multi-center study, 9020 participants aged (2206 non-elderly adults and 6814 elderly adults) from 105 communities of Shanghai were included (Assessment of Somatic Symptom in Chinese Community-Dwelling People, clinical trial number NCT04815863, registered on 06/12/2020). The Somatic Symptom Scale-China (SSS-CN) questionnaire was used to measure SSD. Depressive and anxiety disorders were assessed by the Patient Health Questionnaire-9 (PHQ-9) and Generalized Anxiety Disorder-7 (GAD-7), respectively.

**Results:**

The prevalence of SSD in the elderly was higher than that in the non-elderly (63.2% vs. 45.3%). The elderly suffered more severe SSD (20.4% moderate and severe in elderly vs. 12.0% in non-elderly) and are 1.560 times more likely to have the disorder (95%CI: 1.399–1.739; *p* < .001) than the non-elderly. Comorbidity of depressive or anxiety disorders was 3.7 times higher than would be expected in the general population. Additionally, the results of adjusted multivariate analyses identified older age, female sex, and comorbid physical diseases as predictive risk factors of SSD in the elderly group.

**Conclusions:**

With higher prevalence of common physical problems (including hypertension, diabetes mellitus and cardio/cerebrovascular disease), the elderly in Shanghai are more vulnerable to have SSD and are more likely to suffer from comorbid depressive and anxiety disorders. SSD screening should be given more attention in the elderly, especially among older females with several comorbid physical diseases.

**Supplementary Information:**

The online version contains supplementary material available at 10.1186/s12888-022-03907-1.

## Introduction and objectives

As the world population ages rapidly, elderly adults (over 60 years old, as defined by World Health Organization [1]) now account for 13.5% of the global population [2] and 18.7% of the population of China [3]. With increasing life expectancy, longevity has increased from 66.5 years old in 2006 to 73.3 years old by 2019 [4]. By 2050, the number of aging people is expected to reach 2 billion [5]. However, the successful extension of the human life span raises a deeper demand for quality-of-life improvement, rather than solely extended living time.

Psychological problems are an important factor affecting the quality of life. Published in 2018, the “White Paper on Mental Health of Chinese Urban Residents” surveyed 1.13 million people from 556 medical institutions (health examination centers and general hospitals) on physical and psychiatric questions [6]. Based on these responses, 73.6% of urban residents were considered to have suboptimal mental health [6], indicating that psychological problems have become an inevitable hindrance to healthy development.

Since the elderly have grown into a larger entity, a special focus on the older population is needed. Compared with the non-elderly, the elderly population has unique social and biological characteristics. Generally, the elderly have a higher prevalence of hypertension [7], diabetes [8], and cardio/cerebrovascular (CV) diseases [9]. Their paces of life, occupational stressors, and marital statuses are different from those of non-elderly individuals. Therefore, this group may also have a different prevalence of somatic symptom disorder (SSD), depressive disorders, and anxiety disorders. While previous literature has investigated depressive or anxiety disorders in aged populations [10–13], SSD in the elderly has rarely been addressed. In a European study, SSD prevalence in the elderly was reported to range from 0–13.5%, based on the Diagnostic and Statistical Manual of Mental Disorders (DSM‐IV) criteria [14–18]. For example, Dehoust et al. reported that, overall, 3.88% of elderly participants met the criteria for any past‐year somatoform disorder based on the multi-center MentDis_ICF65 + study [18]. However, the diagnostic criteria for somatization disorder defined in the DSM‐IV have frequently been criticized as overly restrictive. Therefore, the DSM-5, which emphasizes broader concepts, now defines SSD as “symptoms that are difficult to explain after adequate evaluation; even when a significant medical disease is present.”

To the best of our knowledge, there is no research comparing the prevalence of SSD, depressive disorders, and anxiety disorders between elderly and non-elderly populations. Current data are not sufficient to determine the prevalence of SSD under DSM-5 criteria. As the clinical relevance of SSD in the elderly is uncertain, these studies are urgently required for the optimal allocation of medical resources and health management of elderly community members.

To screen for SSD, we designed a prospective cross-sectional study to assess physical and psychological characteristics in elderly people from 105 communities in Shanghai, China. Differs from SSD-12 (focuses mainly on psychological feelings, which views somatic discomfort as a general concept, and focuses on the impact of the disease on the individual), we previously developed the Somatic Symptom Scale-China (SSS-CN) questionnaire, a somatic and psychological symptom scale based on the DSM-5 that can be used to assess a combination of psychological, behavioral, and somatic symptoms and which is designed to assess both presence and severity of symptoms [19]. We validated the reliability and validity of this instrument in a previous study [20]. The Patient Health Questionnaire-9 (PHQ-9) and Generalized Anxiety Disorder Screener (GAD-7) were used to evaluate depressive and anxiety disorders, respectively. The purpose of our study was threefold: (1) to clarify the prevalence of SSD, depressive disorders, and anxiety disorders in the elderly in China; (2) to identify physical and psychological differences between the elderly and non-elderly; and (3) to explore risk factors for SSD in the elderly.

## Materials and methods

### Study design and participants

This prospective cross-sectional study (Assessment of Somatic Symptom in Chinese Community-Dwelling People, ClinicalTrials.gov identifier NCT04815863, registered on 06/12/2020) is a multicenter registry conducted in Shanghai, China, under the supervision of the Shanghai Association of Chinese Integrative Medicine.

A multistage and stratified systematic sampling technique was used to choose representative districts from sixteen districts in Shanghai, which were then divided into low, medium, and high economic levels according to the per capita gross domestic product (GDP) of each district in 2018. Eleven districts were selected proportionally and randomly and represented by 105 community health service institutions. Inclusion criteria were (a) voluntary participation and provision of written consent to participate in the study’s evaluation and assessment and (b) completion of the SSS-CN questionnaire, Patient Health Questionnaire-9 (PHQ-9) and Generalized Anxiety Disorder-7 (GAD-7) questionnaires. Exclusion criteria were (a) patients lacking self-assessed abilities or who refused to participate, (b) patients who have been previously confirmed to have serious mental disorders, mental retardation, or dementia, (c) patients with cancer or central nervous system diseases, (d) patients with any missing data within questionnaire items or with more than one item missing from sociodemographic information. The study was approved by institutional review committees at each center. The study is compliant with the 1975 Declaration of Helsinki guidelines.

### Definition of the elderly

Elderly populations were defined according to the World Health Organization recommendations [1]. People over age 60 are considered elderly in developing countries. In our study, participants were divided into two age groups: elderly (≥ 60 years) and non-elderly (18–60 years).

### Description of SSS-CN and assessment of SSD

The SSS-CN questionnaire is self-administered with an abbreviated 20-item measure. Briefly, it is composed of four dimensions: physical disorders, depressive disorders, anxiety disorders and depressive and anxiety disorders. Half of the items ask about physical complaints (one item per body system). We validated the reliability and validity of this instrument in a previous study [21]. The total score can range from 20 to 80, and test–retest reliability was 0.9. To obtain accurate cutoff values in this group of populations, we randomly selected 202 participants who undertook both questionnaires to be interviewed by a physician team. The physician teams (physicians qualified as national psychological counsellors or psychologists) diagnosed SSD based on DSM-5 criteria. These physician assessments were used as the reference standard, and a cutoff value of 30 yielded an optimal sensitivity of 0.96 and specificity of 0.86. These findings were consistent with the research of Jiang et al. [19]. Thus, SSS-CN scores ranging from 20 to 29, 30–39, 40–59 and ≥ 60 correspond to normal, mild, moderate and severe SSD, respectively. The SSS-CN is attached in the Supplementary material.

### Assessment of depressive and anxiety disorders

All participants were assessed for depressive and anxiety disorders according to the PHQ-9 and GAD-7, which evaluate the frequencies at which certain symptoms had been experienced over the last two weeks, ranging from 0 (‘not at all’) to 3 (‘nearly every day’). These questionnaires were self-administered. The cutoff values are listed below. For PHQ-9, scores of 5–9 indicate mild depressive disorder, 10–14 indicate moderate depressive disorder, 15–19 indicate moderately severe depressive disorder and ≥ 20 indicate severe depressive disorder [22]. For GAD-7, scores range from 0 to 21, with scores of 5–9, 10–14, and ≥ 15 representing mild, moderate, and severe anxiety symptom levels, respectively [23].

### Sociodemographic and clinical characteristics

Sociodemographic data, including age, gender, level of education and marital status, were also collected. A semi-structured questionnaire concerning clinical characteristics was administered to survey participants’ medical and medication histories.

### Data collection and management

The survey was conducted by trained surveyors. A community pilot study was conducted, and surveyors developed unified explanations of each item based on this pilot study to ensure sufficient understanding. Native Chinese language was used. Surveyors read the items and helped to fill out the questionnaires if the participants were illiterate. Surveyors and supervisors were trained to understand questionnaire contents and study procedures. Data were collected by trained clinical researchers, and data collection was supervised by specialist mental health workers.

Data were entered into the EpiData 3.1 (EpiData for Windows; EpiData Association, Odense, Denmark) database using dual data entry. Over 10% of the database was further audited, including logical consistency checks and extraction of information from participants’ community health records to ensure accuracy and completeness. A unique identifier number was assigned to each participant. Proper categorization and coding of data were performed during data cleaning phases. Database access was password-protected, and access was restricted to key team personnel.

### Statistical analyses

Descriptive statistics for each group, such as counts and percentages (%) for non-continuous variables, were reported for all sociodemographic and clinical characteristics. The prevalences of SSD of various severities for different genders and for participants diagnosed with depressive and anxiety disorders were calculated.

In order to assess whether SSD, depressive and anxiety disorders are associated with aged groups and whether depressive and anxiety disorders are associated with the severity of SSD, binary logistic regressions were performed, adjusting for gender, educational level (middle school and below, high school, college, master and above), marital status (never married, married, divorced, widowed), hypertension (yes/no), diabetes mellitus (yes/no), CV disease (yes/no), endocrine and metabolic disease except DM (yes/no), other diseases (yes/no), surgery (yes/no) and antipsychotic intake (yes/no) in all models.

Univariate analyses and binary logistic regression models were used to assess the effects of sociodemographic factors and clinical characteristics on SSD in the non-elderly and elderly. Categorical sociodemographic and clinical variables were analyzed by chi-square test. Binary logistic regressions were performed using SSD (yes/no) as the dependent variable, adjusting for gender, educational level, marital status, and antipsychotic intake. Odds ratios (ORs), 95% confidence interval (95%CI) values, and significance values (P-values) were reported in tabular form and forest plots. The number of risk factors for each participant was calculated to assess the association between the number of risk factors and SSD prevalence. Subgroup analysis by gender in the elderly group was also conducted.

Fisher’s exact test was used instead of the chi-square test only when one or more expectations for each cell of the 2-by-2 tables was below 5. A significance level was set at a *p*-value < 0.05 (two-tailed). Missing data were imputed with a multiple imputation approach. All statistical analyses were performed with SPSS 22 (IBM SPSS Statistics for Windows, Version 22.0. Armonk, NY: IBM Corp).

## Results

### Study population

Eleven thousand two hundred community dwellers people between March 2019 and June 2019 were approached, and the rejection rate was less than 1%. Then, all collected questionnaires were checked for completeness and quality by trained surveyors based on inclusion criteria and exclusion criteria. Based on inclusion and exclusion criteria, 9020 subjects between 18 to 100 were included in this prospective multicenter registry, composed of 6814 elderly (age ≥ 60 years) and 2206 non-elderly participants (18–59 years). The baseline characteristics of study participants are presented in Table 1. The median age of elderly participants was 70 (interquartile range, 65- to 75-year-old), and the median age for non-elderly participants was 48 (interquartile range, 36- to 56-year-old). Elderly participants had a higher prevalence of physical diseases and a lower average educational level.

**Table 1:** Sociodemographic and clinical features

Subgroup	Non-Elderly n(%)	Elderly n(%)	*X*^*2*^	*P* Value
**Gender**			28.432	< .001
Male	693(31.5)	2567(37.8)		
Female	1509(68.5)	4231(62.2)		
**Education**			504.549	< .001
Middle school and below	670(30.4)	3481(51.2)		
High school	617(28.0)	2010(29.6)		
College	859(39.1)	1262(18.5)		
Master and above	56(2.5)	45(0.7)		
**Marital status**			36.605	< .001
Never married	172(7.8)	307(4.5)		
Married	1842(83.7)	5868(86.4)		
Divorced	23(1.0)	90(1.3)		
Widowed	165(7.5)	533(7.8)		
**Hypertension**			807.174	< .001
Yes	409(18.5)	3621(53.1)		
No	1797(81.5)	3193(46.9)		
**Diabetes mellitus**			280.303	< .001
Yes	190(8.6)	1731(25.4)		
No	2016(91.4)	5083(74.6)		
**Cardio/Cerebrovascular disease**			418.438	< .001
Yes	154(7.0)	1910(28.0)		
No	2052(93.0)	4904(72.0)		
**Endocrine and metabolic disease except DM**			3.899	.048
Yes	14(0.6)	76(1.1)		
No	2192(99.4)	6738(98.9)		
**Other diseases**			2.420	.120
Yes	29(1.3)	123(1.8)		
No	2177(98.7)	6691(98.2)		
**Surgery**			16.224	< .001
Yes	4(0.2)	75(1.1)		
No	2202(99.8)	6739(98.9)		
**Antipsychotics**			34.016	< .001
Yes	8(0.4)	154(2.3)		
No	2198(99.6)	6660(97.7)		

### Prevalence and severity of SSD

**Fig. 1:**
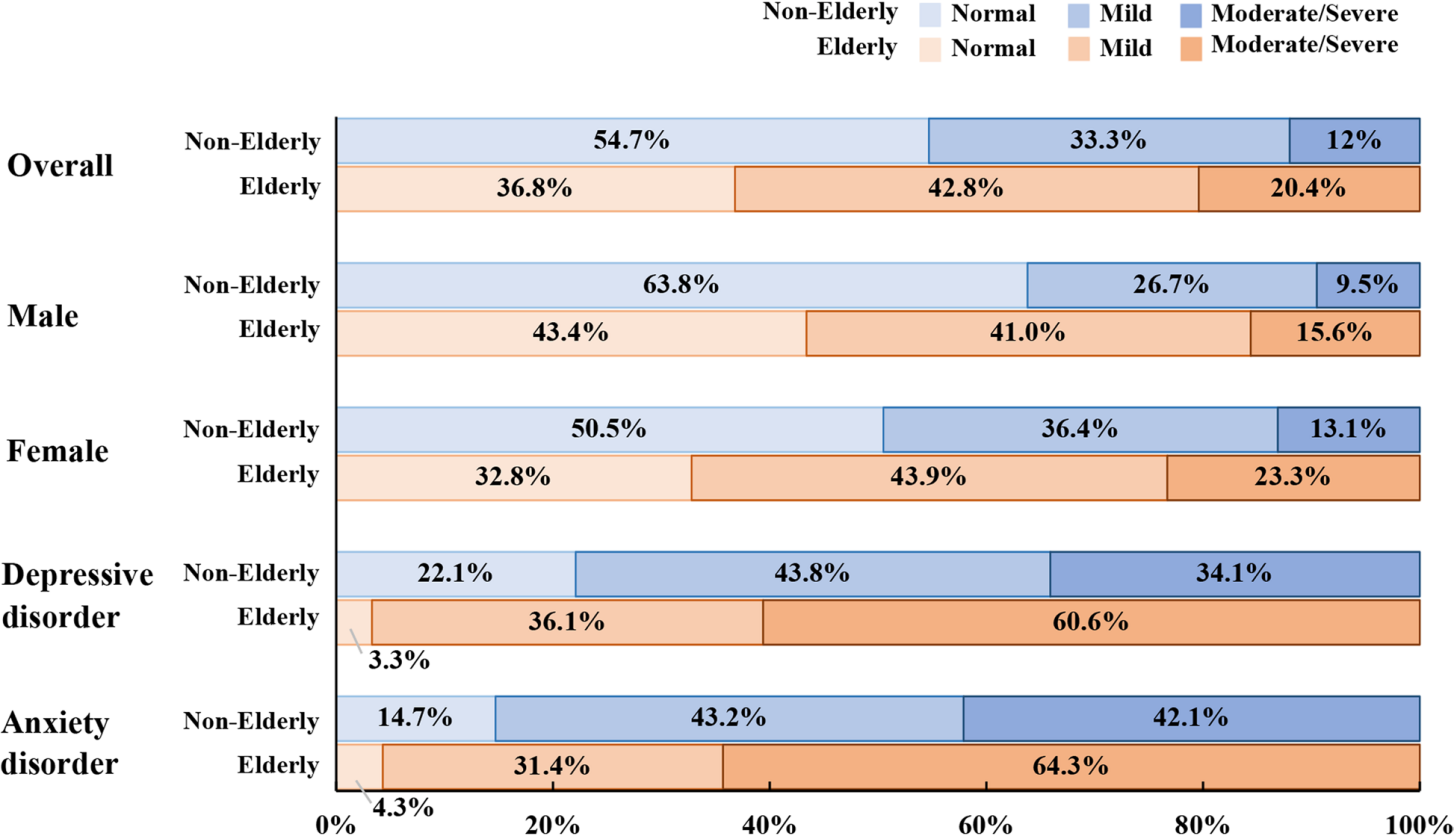
Severity comparison of SSD between non-elderly and elderly populations

**Fig. 2:**
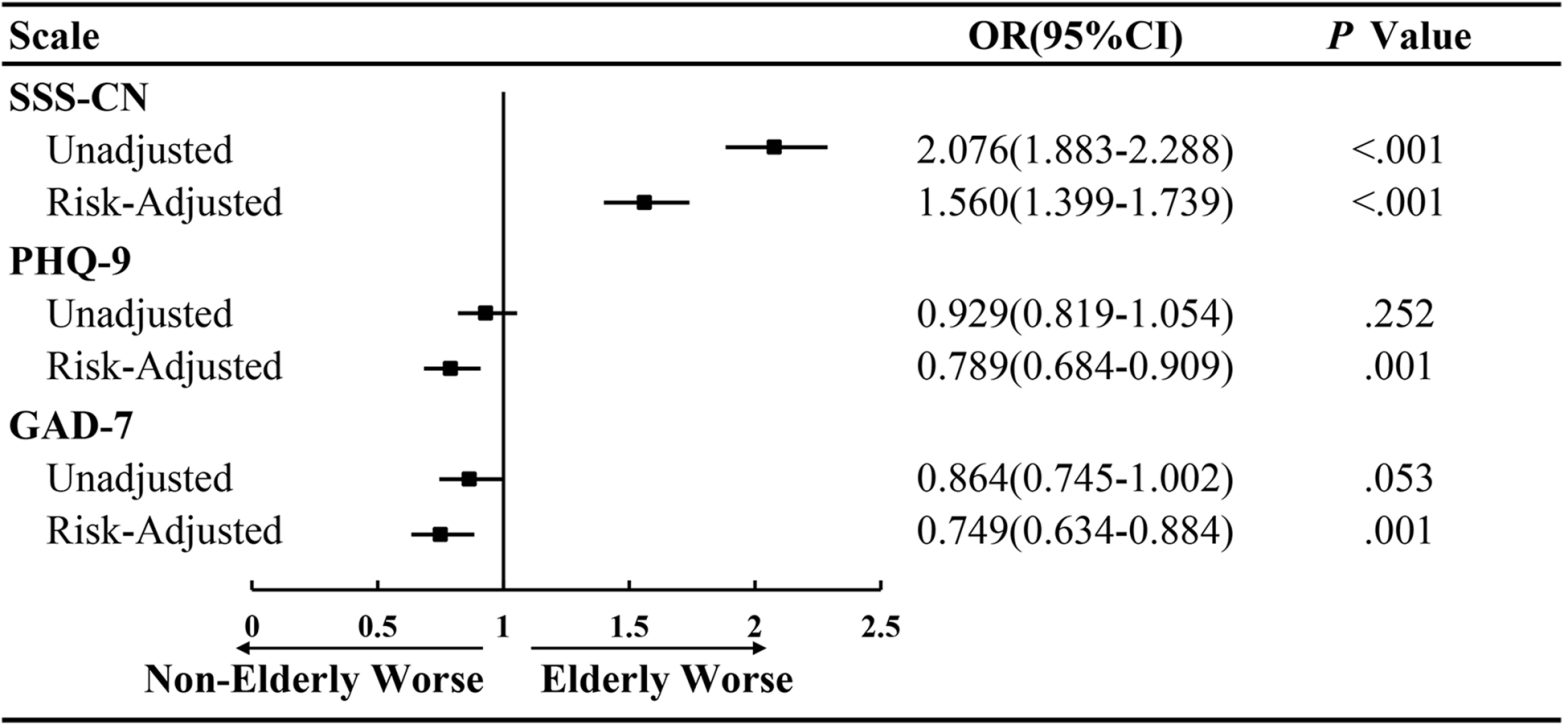
Odds ratios (OR) of SSD, depressive disorder, and anxiety disorder between non-elderly and elderly groups. The non-elderly group was set as the reference category (Ref. = 1). Gender, educational level, marital status, hypertension, diabetes mellitus, cardio/cerebrovascular disease, other diseases, and intake of antipsychotics were adjusted

Figure 1 shows the distributions of SSD of different severities in non-elderly and elderly groups. Across all participants, the prevalence of SSD in the elderly (42.8% mild and 20.4% moderate/severe) was higher than that in the non-elderly (33.3% mild and 12% moderate/severe). The percentage of moderate/severe SSD in the elderly was higher (20.4%) than in the non-elderly (12.0%). As shown in Fig. 2, binary logistic regression analyses revealed that the elderly have a higher chance of suffering from SSD after adjustment for comorbid physical diseases and other confounding factors (OR = 1.560; 95%CI: 1.399–1.739; *p* < 0.001). Furthermore, we calculated the prevalence of SSD separately for participants with (*n* = 1865) and without (*n* = 7155) depressive or anxiety disorders, and the prevalence of SSD in patients with combined depressive or anxiety disorders was 3.7 times higher than would be expected in the general population. In elderly participants with SSD, 96.7% exhibited accompanying depressive disorders, and 95.7% exhibited anxiety (shown in Fig. 1, last 2 panels). Elderly participants had a higher percentage of depressive or anxiety disorders compared to the non-elderly group, in addition to exhibiting more severe symptoms.

### Association of SSD with depressive and anxiety disorders

In our study, the rates of depressive disorder and anxiety disorders were 17.0% (*n* = 1535) and 11.2% (*n* = 1014). In the elderly, the prevalence of anxiety disorder was 10.9% (*n* = 741), compared to 12.4% (*n* = 273) in the non-elderly. For depressive disorders, these values were 16.8% (*n* = 1142) and 17.8% (*n* = 393), respectively. Supplementary Table S1 shows the distribution of depressive and anxiety disorders of different severity. After adjusting for confounding factors, elderly populations were less likely to have depressive disorders than non-elderly populations (OR = 0.789, 95%CI: 0.684–0.909; *p* = 0.001) and were also less likely to have anxiety disorders (OR = 0.749; 95%CI: 0.634–0.884; *p* = 0.001) (shown in Fig. 2).

**Fig. 3:**
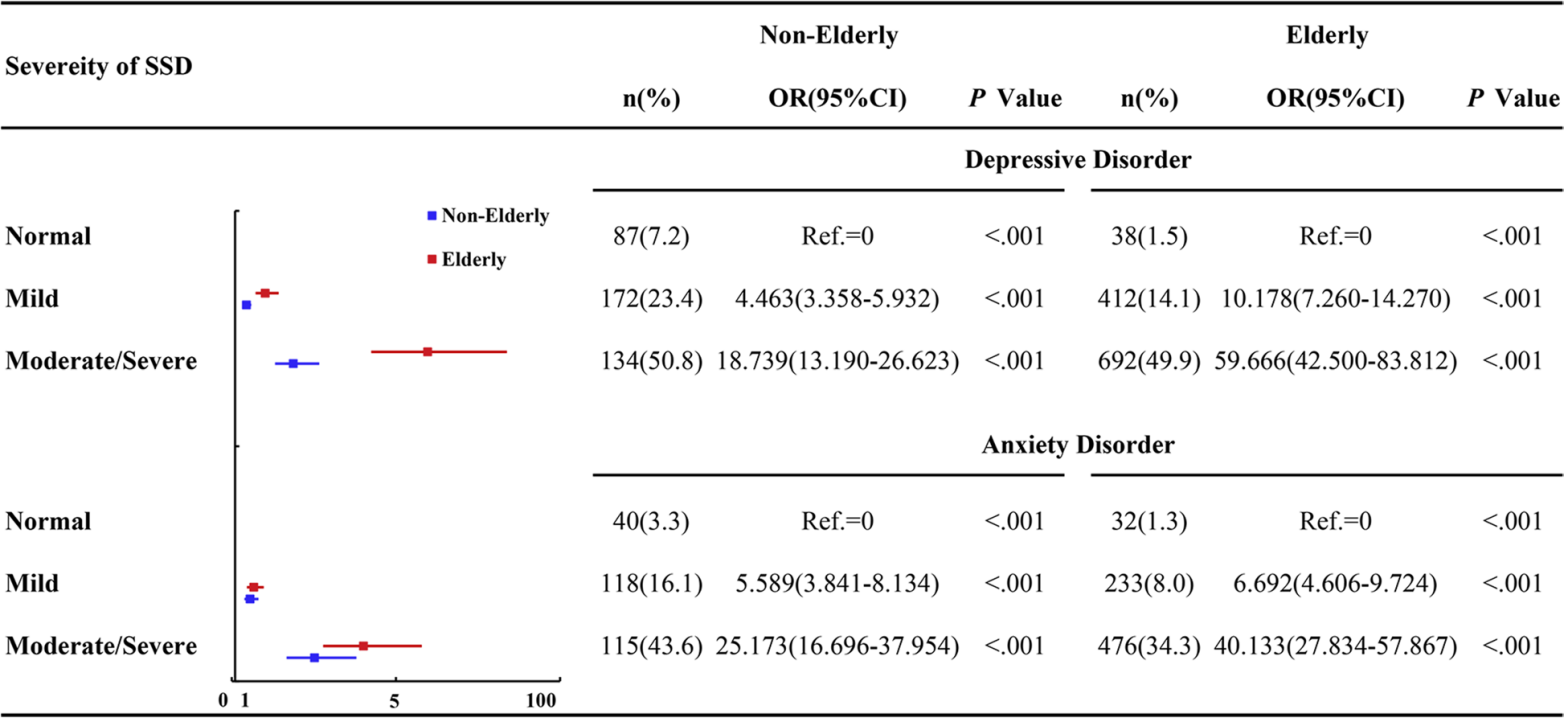
Odds ratio (OR) of mental disorders according to SSD severity. No SSD was set as the reference category (Ref. = 1). Gender, educational level, marital status, hypertension, diabetes mellitus, cardio/cerebrovascular disease, other diseases, and intake of antipsychotics were adjusted

Although the elderly was not prone to anxiety or depressive disorders, binary logistic regression analyses revealed that comorbid SSD increased the risk of depressive and anxiety disorders, even after adjustment for comorbid physical diseases and other confounding factors. Regardless of SSD severity, elderly adults with SSD were at a much higher risk for depressive disorders and anxiety disorders compared to non-elderly adults (shown in Fig. 3).

### Association of sociodemographic and clinical predictors with SSD

Table 2 depicts baseline univariate analyses of elderly and non-elderly participants based on the presence of SSD. Older age, female sex, and changed marital status were significantly predictive of higher SSD rates in the elderly group (*p* < 0.05). Interestingly, educational level was found to be associated with SSD in the non-elderly, but not in the elderly. With regard to clinical characteristics, cardiac disease and cardiovascular risk factors were significantly associated with SSD in elderly participants (*p* < 0.05).

**Table 2:** Univariate analyses of clinical characteristics and SSD

Subgroup	Non-Elderly(*n* = 2206)				Elderly(*n* = 6814)			
	**SSD**	**Non-SSD**	***X***^**2**^	***P* Value**	**SSD**	**Non-SSD**	***X***^***2***^	***P* Value**
**Gender**			33.816	< .001			76.701	< .001
Male	251(36.2)	442(63.8)			1453(56.6)	1114(43.4)		
Female	747(49.5)	762(50.5)			2842(67.2)	1389(32.8)		
**Age group**			-	-			39.444	< .001
60–69	-	-			1964(59.6)	1331(40.4)		
70–79	-	-			1720(65.6)	900(34.4)		
≥ 80	-	-			623(69.3)	276(30.7)		
**Education**			18.415	< .001			4.793	.188
Middle school and below	312(46.6)	358(53.4)			2233(64.1)	1248(35.9)		
High school	316(51.2)	301(48.8)			1234(61.4)	776(38.6)		
College	350(40.7)	509(59.3)			797(63.2)	465(36.8)		
Master and above	20(35.7)	36(64.3)			31(68.9)	14(31.1)		
**Marital status**			1.638	.651			15.136	.002
Never married	71(41.3)	101(58.7)			192(62.5)	115(37.5)		
Married	837(45.4)	1005(54.6)			3666(62.5)	2202(37.5)		
Divorced	11(47.8)	12(52.2)			67(74.4)	23(25.6)		
Widowed	79(47.9)	86(52.1)			370(69.4)	163(30.6)		
**Hypertension**			40.444	< .001			40.384	< .001
Yes	243(59.4)	166(40.6)			2415(66.7)	1206(33.3)		
No	756(42.1)	1041(57.9)			1892(59.3)	1301(40.7)		
**Diabetes mellitus**			33.488	< .001			54.448	< .001
Yes	124(65.3)	66(34.7)			1222(70.6)	509(29.4)		
No	875(43.4)	1141(56.6)			3085(60.7)	1998(39.3)		
**Cardio/Cerebrovascular disease**			55.192	< .001			307.888	< .001
Yes	114(74.0)	40(26.0)			1521(79.6)	389(20.4)		
No	885(43.1)	1167(56.9)			2786(56.8)	2118(43.2)		
**Endocrine and metabolic disease except DM**			0.126	.722			6.875	.009
Yes	7(50.0)	7(50.0)			59(77.6)	17(22.4)		
No	992(45.3)	1200(54.7)			4248(63.0)	2490(37.0)		
**Other diseases**			8.728	.003			17.632	< .001
Yes	21(72.4)	8(27.6)			100(81.3)	23(18.7)		
No	978(44.9)	1199(55.1)			4207(62.9)	2484(37.1)		
**Surgery**			2.882	.090			3.343	.067
Yes	4(100.0)	0(0.0)			55(73.3)	20(26.7)		
No	995(45.2)	1207(54.8)			4252(63.1)	2487(36.9)		
**Antipsychotic**			7.611	.006			47.229	< .001
Yes	8(100.0)	0(0.0)			138(89.6)	16(10.4)		
No	991(45.1)	1207(54.9)			4169(62.6)	2491(37.4)		

**Fig. 4:**
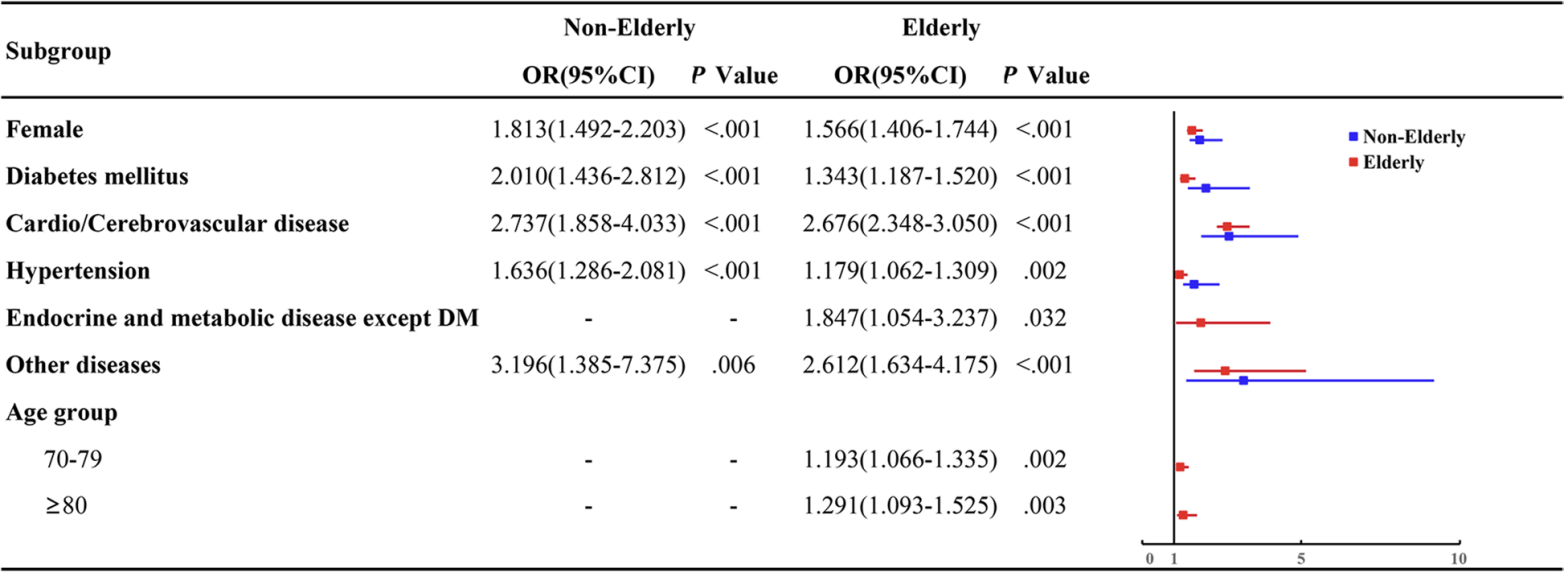
Binary logistic regression models of risk factors of SSD in non-elderly and elderly participants. Male, no hypertension, no diabetes, no cardio/cerebrovascular disease, no other diseases and age 60–69 were set as the reference category (Ref. = 1)

In order to identify risk factors likely to independently predict SSD, the indices in Table 2 were used as input for multivariate analyses (shown in Fig. 4). Older age, female sex, diabetes mellitus, CV disease, hypertension were predictive risk factors of SSD in the elderly group after adjustment for confounding factors. Consideration of age‐related differences revealed that participants between 70–79 years old were 1.193 times more likely to have SSD compared to those between 60–69 years old (95%CI: 1.066–1.335; *p* = 0.002). Similarly, participants over 80 years of age were 1.291 times more likely to have SSD (95%CI: 1.093–1.525; *p* = 0.003). The risk of SSD was 1.566 times higher in elderly females compared to elderly males (95%CI: 1.406–1.744; *p* < 0.001).

**Fig. 5:**
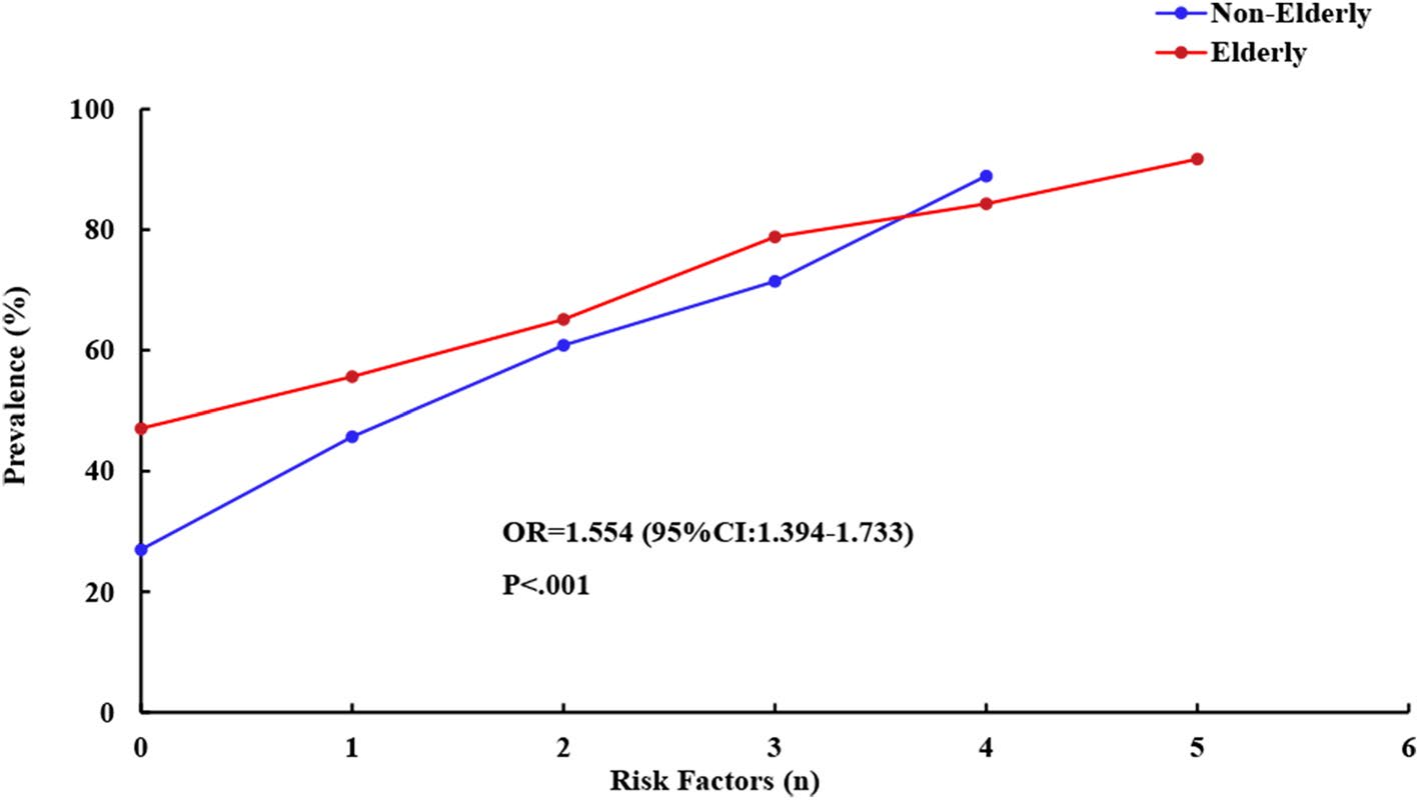
Association of the number of risk factors with SSD prevalence in non-elderly and elderly participants

To explore the influence of risk factors on SSD, the relationship between SSD prevalence and the number of risk factors is shown in Fig. 5. Elderly participants with no risk factors had an SSD prevalence of 47.1% (*n* = 338), and additional risk factors (up to four) were associated with SSD rates of 55.6% (*n* = 1357), 65.1% (*n* = 1400), 78.8%(*n* = 857) and 84.5% (*n* = 355). For non-elderly participants, respective SSD rates by number of patient risk factors were 27.0% (*n* = 131), 45.7% (*n* = 584), 60.8% (*n* = 203), 71.4% (*n* = 65) and 88.9% (*n* = 16). An elevated number of risk factors corresponded with a higher likelihood of SSD. The prevalence of SSD in the elderly was 1.554 times higher than that of the non-elderly group when controlling for the number of risk factors (95%CI: 1.394–1.733; *p* < 0.001). Notably, when the number of risk factors reached four, SSD prevalence in the non-elderly surpassed that observed in the elderly (shown in Fig. 5).

## Discussion

**Fig. 6:**
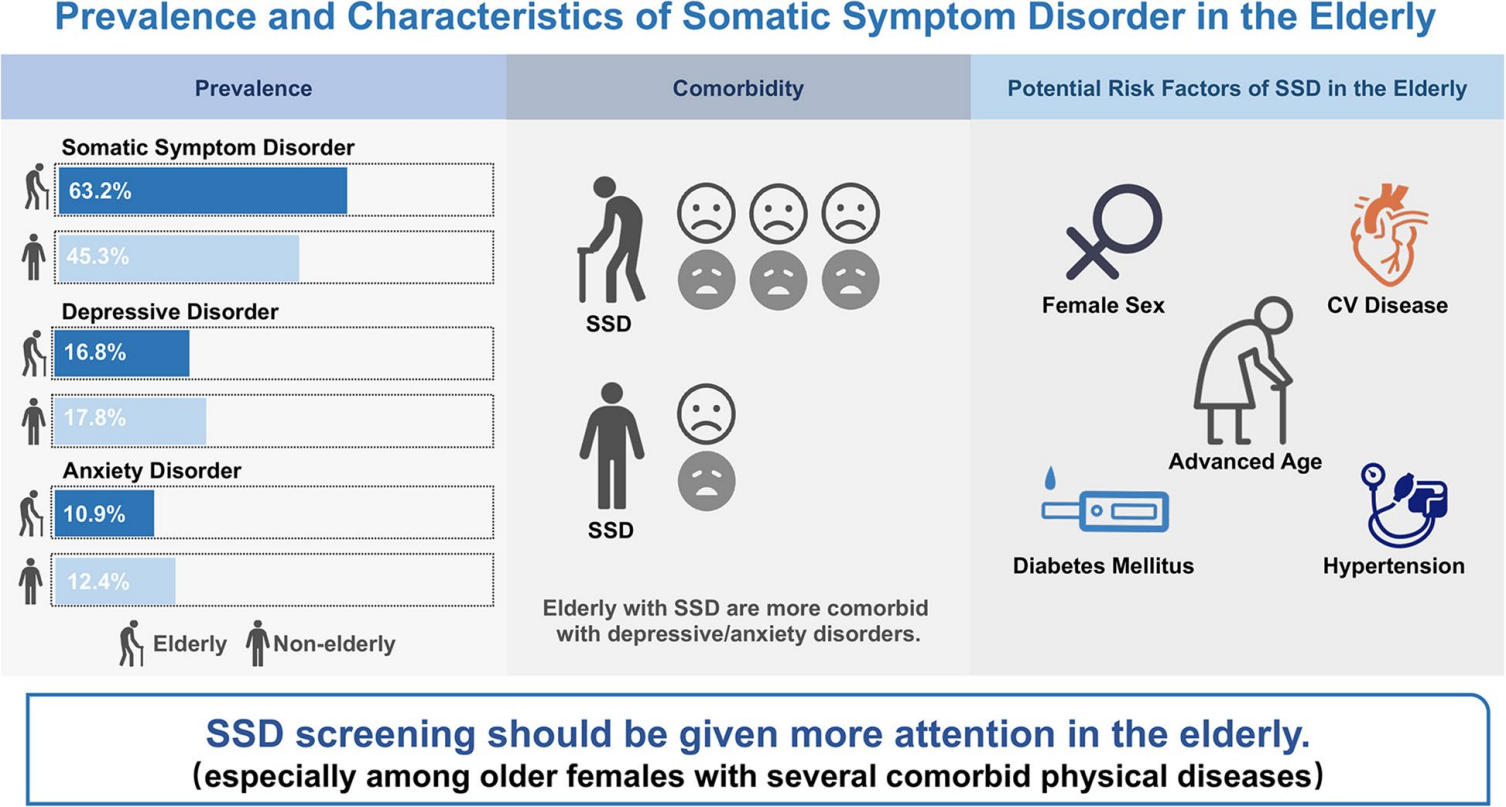
Central illustration of the article

This study was conducted across community health service institutions with majority-elderly participant populations. To our knowledge, our study is the first prospective cross-sectional study focusing on the clinical characteristics of SSD in elderly and non-elderly groups. This study reveals that the prevalence of SSD in the elderly in China is higher than that in non-elderly populations, and SSD severity tends to worsen with age. Additionally, comorbidity of depressive and anxiety disorders among the elderly was more closely related to the prevalence and severity of SSD. Finally, we found that female sex and the existence of physical diseases are associated with higher SSD prevalence (shown in Fig. 6).

### Higher prevalence of SSD among the elderly

The high prevalence of SSD has many detrimental impacts on the elderly. Firstly, somatic symptoms and related disorders are associated with an increased risk for suicidal ideation and suicide attempts, with an estimated 24–34% of participants disclosing current active suicidal ideation and 13–67% of participants disclosing a prior suicide attempt [24]. SSD could heavily impact the lifestyles of elderly patients, decreasing exercising ability and leading to the occurrence of sarcopenia [25]. The psychosocial distress brought by SSD can sometimes even cause serious organic diseases, such as myocardial infarction [26].

Shanghai was selected as the population of interest for this study because its life expectancy is 7 years higher than the national average [27, 28]. Shanghai has a large elderly population in which SSD characteristics can easily be explored. Unlike a previous study by Dehoust et al. [18], which focused only on the elderly, our study sought to identify differences between the elderly and the non-elderly populations. Of note, the prevalence of SSD detected in our study is higher than those reported by other studies [18, 29]. This may be due to the different criteria employed for SSD assessment. SSD from Hilderink study was diagnosed based on DSM-IV. Our questionnaire (SSS-CN), is based on DSM-5. The diagnostic criteria of SSD in the DSM-5 are less restrictive compared with those defined by the DSM-IV. The DSM-5 focuses more on physical symptoms, as well as on excessive thoughts, emotions, and/or behaviors brought on by them, which may result in a higher diagnosis rate for SSD.

Compared with non-elderly participants, we found that the severity of SSD was greater in elderly participants, and the condition became more severe with aging. Several reasons may account for these observations. First, elderly participants typically had more physical diseases, such as hypertension, diabetes, and CV disease, that can induce somatic symptoms. However, Chinese elders tend to have a sense of shame in expressing mental discomfort, leading these experienced to be transduced into the form of bodily discomfort [30]. Second, biologically, dopamine levels decline by around 10% with each additional decade of life, and this reduction is associated with declined mental performance [31]. Serotonin levels also fall with increasing age and may be implicated in the regulation of synaptic plasticity and neurogenesis [32]. These aging-associated changes in neuro-endocrine secretion provide a biological explanation for the high prevalence of SSD in the elderly. Finally, the lack of estrogen in elderly women may increase the occurrence of SSD, as a recent study has suggested that estrogen supplementation can increase dopaminergic responsivity [33] and help improve encephalopathy [34].

### Comorbidity of SSD with depressive and anxiety disorders

Comorbidity of SSD with depressive and anxiety disorders is expected [35–37]. In our study, we identified more SSD patients with depressive or anxiety disorders among the elderly, and the degree of SSD in these patients tended to be more severe than in their non-elderly counterparts. De Vroege et al. found that comorbid depressive and anxiety disorders occurred frequently within patients suffering from SSD (75.1% and 65.7%, respectively), which are much higher than that of our results. This could be due to the population difference. In De Vroege’s study [38], consecutive patients suffering from somatic symptoms and related disorders were enrolled. Patients were aware of their illnesses, more willing to express their psychological discomfort, resulting in higher comorbidity of depressive and anxiety disorders. Our study enrolled community-dwelling people, most of which hadn’t been previously diagnosed with SSD or other mental illnesses. They may not be willing to express their psychological discomfort.

Bernd et al. previously demonstrated that the contribution of commonalities underlying depressive disorder, anxiety disorder and somatization to functional impairment substantially exceeded the contributions of their independent parts [39]. However, no literature existed focusing on these comorbidities among the elderly. Our findings, therefore, fill a critical gap in this field. Recognizing the characteristics of physical and psychological problems found in elderly populations is critical for the future development of pharmaceutical and psychological interventions with appropriate intensity and timing.

### Prevalence and number of risk factors

As expected, SSD occurrence was positively correlated with the number of risk factors, and prevalence was higher in the elderly (shown in Fig. 5). Intriguingly, when the number of risk factors reached four, SSD rates were higher in non-elderly participants. We propose two potential explanations for this phenomenon. Firstly, the number of non-elderly participants with ≥ 4 risk factors (*n* = 18) was far smaller than the number of elderly participants meeting the same criteria (*n* = 420), a potential source of bias. Secondly, non-elderly participants suffering from many (≥ 4) physical diseases may exhibit relatively higher stress levels.

### Implications for early intervention of SSD

Regarding the treatment of SSD, neurocognitive behavioral therapy (CBT) is of choice [40, 41]. However, De Vroege et al. reported high frequency of CBT dropout in patients with SSD and CBT has a limited effect [38]. Our findings provided rationales for the early detection and timely intervention in elderly patients. We found that older females with several comorbid physical diseases were vulnerable subjects. Regular screenings are suggested to be performed on those people. Proper interventions, such as raising social awareness of somatization disorder, involving elderly in more community activities are the possible approaches.

### Strengths and limitations

To our knowledge, this is the first study to investigate the clinical characteristics of SSD in elderly and non-elderly with the largest population. The relevant demographic, clinical features, and risk factors of the elderly with SSD are emphasized. We believe that our findings could provide a reference to economically developed regions in China in dealing with SSD in the elderly and prepare for the more severe aging in the future.

Our study also has several limitations. First, although our study represents the largest elderly and non-elderly population for which SSD has been studied to date, it is, nevertheless, a cross-sectional study for the elderly. No information was obtained about disorder progression or treatment requirements. Secondly, participants in our study were all enrolled from Shanghai, a city with better medical care, better education [27, 28] and more social stress than most other cities. Further research will be required to determine whether our findings can be generalized nationwide.

## Conclusions

Our findings demonstrate that, compared to the non-elderly, the elderly have a higher prevalence of SSD and exhibit more severe comorbidities with physical and psychological problems. Older females with several comorbid physical diseases were identified as an extremely vulnerable group; attention should be given to screening these outpatients.

## Supplementary Information

**Additional file 1.**

## Data Availability

The data that support the findings of this study are not publicly available due to their containing information that could compromise the privacy of research participants but are available from Yongxia Qiao upon reasonable request.

## Abbreviations

SSD: Somatic Symptom Disorder
SSS-CN: Somatic Symptom Scale-China
PHQ-9: Patient Health Questionnaire-9
GAD-7: Generalized Anxiety Disorder-7
