# Supplementary material for: Prevalence and characteristics of somatic symptom disorder in the elderly in a community-based population: a large-scale cross-sectional study in China
Source: BMC Psychiatry. 2022 Apr 12;22:257. doi: 10.1186/s12888-022-03907-1 (PMC9004132; doi:10.1186/s12888-022-03907-1)
Supplement: Supplementary file 1 — Additional file 1. [file 12888_2022_3907_MOESM1_ESM.docx]

**Supplementary Figure S1. The Somatic Symptom Scale-China**


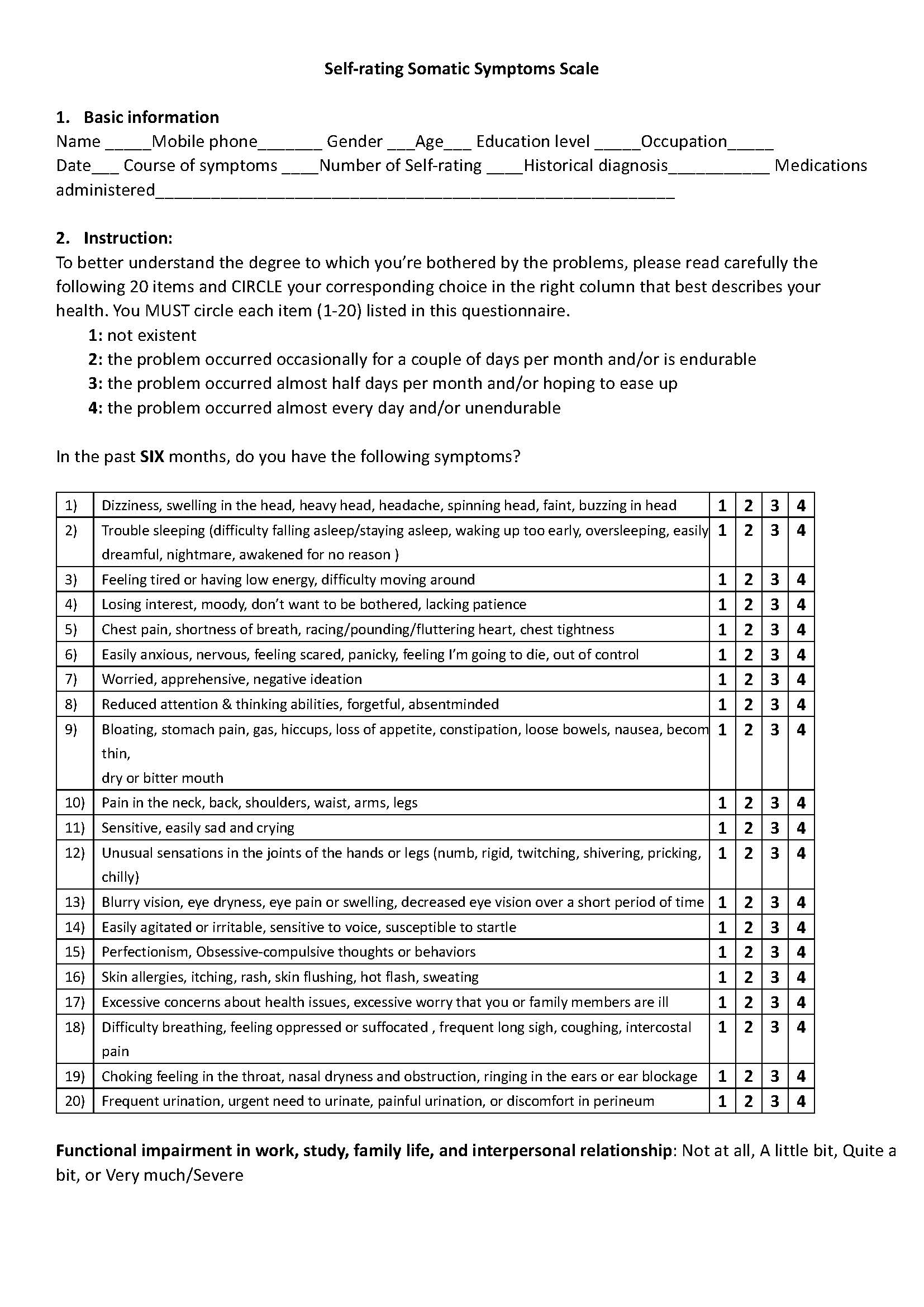


The questionnaire can be found from: Jiang M, Zhang W, Su X, Gao C, Chen B, Feng Z, et al. Identifying and measuring the severity of somatic symptom disorder using the Self-reported Somatic Symptom Scale-China (SSS-CN): a research protocol for a diagnostic study. BMJ Open. 2019;9(9):e024290.

**Supplementary Table S1. Distribution of Depressive and Anxiety Disorders**

| Level of Severity,  Scale Score | Overall(n=9020) n(%) | Non-Elderly(n=2206) n(%) | Elderly(n=6814) n(%) |
| --- | --- | --- | --- |
| Depression (PHQ-9) |  |  |  |
| Normal, 0–4 | 7485(83.0) | 1813(82.2) | 5672(83.2) |
| Mild, 5-9 | 1175(13.0) | 321(14.6) | 854(12.5) |
| Moderate, 10-14 | 262(2.9) | 52(2.4) | 210(3.1) |
| Moderately severe,15-19 | 75(0.8) | 16(0.7) | 59(0.9) |
| Severe, 20-27 | 23(0.3) | 4(0.2) | 19(0.3) |
| Anxiety (GAD-7) |  |  |  |
| Normal, 0–4 | 8006(88.8) | 1933(87.6) | 6073(89.1) |
| Mild, 5-9 | 807(8.9) | 226(10.2) | 581(8.5) |
| Moderate, 10-14 | 147(1.6) | 32(1.5) | 115(1.7) |
| Severe, 15-21 | 60(0.7) | 15(0.7) | 45(0.7) |
